# Comparative Biofunctionality Assessment of Lignin and Lignin/Chitosan Nanoparticles: Impact of Chitosan Co-Assembly on Cytotoxicity, Cytocompatibility, Radical-Scavenging Activity, and Antimicrobial Performance

**DOI:** 10.3390/pharmaceutics18030350

**Published:** 2026-03-11

**Authors:** Tsvetelina Zagorcheva, Boika Andonova-Lilova, Denitsa Georgieva, Silviya Hristova, Zhani Yanev, Nikolina Rusenova, Georgi Beev, Kamelia Petkova-Parlapanska, Galina Nikolova, Yanka Karamalakova, Zvezdelina Yaneva

**Affiliations:** 1 Agrobiotechnology Department, AgroBio Institute, 8 Dragan Tzankov Blvd., 1164 Sofia, Bulgaria; tzvetelina.zagorcheva@gmail.com (T.Z.); boika_andonova@hotmail.com (B.A.-L.); 2 Research & Development & Innovation Consortium, 1784 Sofia, Bulgaria; 3 Department of Pharmacology, Animal Physiology, Biochemistry and Chemistry, Faculty of Veterinary Medicine, Trakia University, 6000 Stara Zagora, Bulgaria; denitsa.georgieva@trakia-uni.bg (D.G.); silviya.hristova@trakia-uni.bg (S.H.); 4 Faculty of Industrial Technology, Technical University of Sofia, 1756 Sofia, Bulgaria; zhani@abv.bg; 5 Department of Veterinary Microbiology, Infectious and Parasitic Diseases, Faculty of Veterinary Medicine, Trakia University, 6000 Stara Zagora, Bulgaria; nikolina.rusenova@trakia-uni.bg; 6 Department of Biological Sciences, Faculty of Agriculture, Trakia University, 6000 Stara Zagora, Bulgaria; georgi.beev@trakia-uni.bg; 7 Department of Chemistry and Biochemistry, Faculty of Medicine, Trakia University, 6000 Stara Zagora, Bulgaria; kamelia.parlapanska@trakia-uni.bg (K.P.-P.); galina.nikolova@trakia-uni.bg (G.N.); yanka.karamalakova@trakia-uni.bg (Y.K.)

**Keywords:** lignin/chitosan nanoparticles, cytotoxicity, antioxidant activity, antimicrobial potential, regenerative capacity

## Abstract

**Background/Objectives:** The aim of the present study was to conduct a systematic in vitro assessment of the biofunctionalities of newly synthesized lignin (LNPs) and lignin–chitosan nanoparticles (LCNPs) via a comparative in vitro estimation of their cytotoxicity, cytocompatability potential, radical-scavenging activity, and antimicrobial performance, thereby establishing a benchmark for their sustainable design and biomedical applications. **Methods:** LNPs and LCNPs were synthesized via “green” self-assembly and co-assembly methods. **Results:** In vitro cytotoxicity studies on L929 fibroblasts and HaCaT keratinocytes demonstrated higher long-term viability for LCNPs (half-maximal inhibitory concentration IC_50_ = 3.05 mg/mL at 72 h) compared with LNPs (IC_50_ = 1.37 mg/mL), while both formulations maintained >76% viability at a concentration of 0.5 mg/mL. Electron Paramagnetic Resonance (EPR) and spectrophotometric antioxidant assays displayed strong radical scavenging activity, with LNPs excelling in ^●^OH, NO, and ABTS scavenging and LCNPs exhibiting enhanced lipid peroxidation and superoxide inhibition potential. Antimicrobial testing revealed minimal inhibitory concentration (MIC) reductions of the nanoparticles up to 8–13-fold compared to lignin solutions, with LCNPs showing higher activity against Gram-positive and Gram-negative microbial strains. **Conclusions:** These results highlight LCNPs as biocompatible, antioxidant, and antimicrobial nanoplatforms with potential for regenerative medicine, oxidative stress mitigation, and infection control.

## 1. Introduction

Nanoparticle-based delivery systems have become pivotal in modern biomedicine, offering enhanced targeting, controlled release, and theranostic capabilities, including simultaneous therapeutic and diagnostic functions [1,2]. In this respect, there is a growing demand for environmentally friendly and biocompatible “green” micro- and nanocarriers that can scavenge ROS, reduce systemic toxicity, and improve therapeutic outcomes [1,2,3,4,5].

Among naturally derived polymers, lignin has emerged as a versatile biopolymer for nanoparticle synthesis due to its structural complexity, abundance, and multifunctional properties. Lignin is a semi-random, three-dimensional aromatic heteropolymer composed of phenylpropanoid units linked via ether (Ar-O-Ar’) and carbon–carbon (C-C) bonds [1,6,7]. Its phenolic, aliphatic -OH, and methoxy (-OCH_3_) groups confer inherent antioxidant, antimicrobial, UV-protective, antiviral, anti-inflammatory, and anticoagulant properties while enabling chemical modification and physical self-assembly into the structures of nanoscale carriers [6,7,8,9,10,11]. The -OH groups at the α- and γ- positions of the phenolic chains enhance antibacterial activity by disrupting bacterial cell membranes and interfering with nucleic acid synthesis [8,9,12]. Incorporation of bioactive compounds such as flavonoids (e.g., morin, quercetin, naringenin, etc.) further improves antibacterial efficacy, offering a synergistic platform for biomedical applications [9,13,14].

Chitosan, the deacetylated derivative of chitin, complements lignin by providing a cationic, biodegradable, and biocompatible polymer matrix with intrinsic antimicrobial, antioxidant, antiproliferative, and tissue-regenerative properties [7,9,15]. Its positive charge allows electrostatic co-assembly with the negatively charged lignin moieties to form lignin–chitosan nanoformulations with enhanced colloidal stability, mechanical strength, and multifunctionality compared with either polymer alone [9,15,16,17]. Lignin–chitosan-based nanoparticles (LCNPs) can be engineered into pH-responsive core-shell architectures, enabling selective drug release in acidic tumor microenvironments or infected wounds, thereby achieving stimuli-sensitive therapeutic delivery [18,19].

Recent studies have leveraged microfluidic synthesis and interfacial co-assembly techniques to reproducibly produce LCNPs with narrow size distributions, high encapsulation efficiency, and controlled release profiles for both hydrophilic and hydrophobic drugs and naturally derived bioactive substances [15,16]. Lignin/chitosan composites offer controlled release properties suitable for cancer therapy, ensuring sustained and targeted delivery of therapeutic agents [6,20]. Chai et al. reported the synthesis of pH-responsive lignin/chitosan nanoparticles to deliver the anticancer drugs docetaxel and curcumin, exhibiting satisfactory drug loading efficiency, biocompatibility, and pH-responsive release [21]. Furthermore, biocompatible luminescent nanoparticles, such as those doped with samarium, provided enhanced contrast for bioimaging, improving drug delivery applications [6]. These systems demonstrated enhanced cytotoxicity against cancer cells while maintaining favorable cytocompatibility with non-malignant cells, highlighting their potential for selective anticancer therapy [22,23]. Moreover, functionalization of the nanoformulations enabled theranostic applications, providing bioimaging capabilities, enhanced antimicrobial activity, and additional anticancer effects [10,23,24]. LCNPs and their derivatives were also applied in wound healing and tissue engineering and exhibited remarkable antioxidant activity [16]. Lignin/chitosan films, multilayers, and hydrogels exhibited a broad spectrum of antimicrobial activity, barrier properties, and improved physicochemical stability for applications in food packaging, textiles, and environmental remediation, without demonstrating cytotoxic effects on mammalian cells or zebrafish in vivo [8,9,25,26,27,28,29]. Biocompatible hydrogels prepared by mixing an aqueous-acidic solution of chitosan with alkali lignin did not display toxicity. They did provide a conducive surface for cell attachment and proliferation, making them suitable for application as scaffolds in tissue engineering [30,31]. Pete et al. reported that chitosan-coated lignin nanoparticles could be applied to increase the presence and growth of alkane-degrading microbes at oil-water interfaces [19].

Despite these advances, cytotoxicity and biocompatibility remain critical challenges for clinical translation of lignin/chitosan-based nanoscale formulations. IC_50_ values reported for lignin, chitosan, and LCNPs vary depending on the polymer’s source, nanoparticle size, surface chemistry, and preparation method, emphasizing the need for standardized in vitro assays and in vivo biocompatibility testing [1,2,11]. Comprehensive studies integrating cytotoxicity, regenerative potential, ROS-scavenging capacity, and antimicrobial efficacy for newly synthesized lignin and LCNPs are limited, despite their significance to rational design and clinical translation.

The latter fact, combined with the rapidly evolving scientific appeal for multifunctional, sustainable “green” nanomaterials with broad applications in drug delivery, wound healing, antimicrobial therapies, tissue engineering, and theranostics, provoked the present study. It aims to fill the gap by conducting a systematic assessment of the biofunctionalities of newly synthesized lignin (LNPs) and lignin–chitosan nanoparticles (LCNPs) via a comparative estimation in vitro of their cytotoxicity, cytocompatability potential, radical-scavenging activity, and antimicrobial performance, thereby establishing a benchmark for their sustainable design and biomedical applications.

## 2. Materials and Methods

### 2.1. LNPs and LCNPs Synthesis and Physicochemical Characterization

LNPs were synthesized using an ethanol antisolvent method, proposed by Yaneva et al. [32] (Supplementary Materials Methods S3). LCNPs were synthesized by a method proposed by Yanev et al. comprising solvent/antisolvent precipitation and self-co-assembly [33] (Supplementary Materials Methods S3). The FTIR spectra of LNPs and LCNPs within the wavelength range 400–4000 cm^−1^ were obtained via the KBr disc technique on a TENSOR 37 Bruker FTIR spectrometer (Bruker Optik GmbH, Baden-Württemberg, Germany). TEM imaging was conducted at high resolution on an HR-STEM JEOL JEM-2100 transmission electron microscope (JEOL Ltd., Tokyo, Japan) equipped with a GATAN Orius 832 SC1000 CCD camera (Gatan GmbH, Munich, Germany). The structure of both types of nanoparticles was characterized using an X-ray diffraction (XRD) instrument (PANalytical Empyrean CuKα = 0.15406 nm, 40 kV, 40 mA). The ζ-potential and nanoparticle sizes were measured on a Malvern particle analyzer at 25 °C (Table 1). Each measurement was performed in triplicate to ensure reproducibility.

### 2.2. Cytotoxicity Studies

The mouse connective tissue fibroblast cell line L929 is part of the cell culture collection of the In Vitro Laboratory for Evaluation of Biological Activity and Toxicity, Research and Development and Innovation Consortium in Sofia, Bulgaria. The L929 cell line (ATCC CCL-1) was cultured in Roswell Park Memorial Institute medium (RPMI) (Gibco^TM^, Thermo Fisher Scientific, Waltham, MA, USA) supplemented with 10% fetal bovine serum (FBS) (Gibco^TM^, Thermo Fisher Scientific, Waltham, MA, USA), 1% penicillin, and streptomycin (Sigma-Aldrich Gmbh, Wien, Austria). The cells were maintained in a Panasonic HEPA Class 100 IncuSafe MCO incubator (Panasonic Healthcare Co., Ltd., Etten-Leur, The Netherlands) at 37 °C in a humidified atmosphere containing 5% CO_2_. To investigate the effect of lignin–chitosan nanoparticles on the bio- and cytocompatibility of the cultured cell line L929, cells were seeded in a 96-well plate at a density of 10,000 cells per well. After 24 h of incubation, the cells were ready for nanoparticle treatment and the MTT test.

Studies of the biological activity of the nanoparticles were conducted using the MTT assay (3-(4,5-dimethyltetrazolyl-2-yl)-5-(diphenyltetrazolium-1-yl)-2H-tetrazolium bromide, C_18_H_16_BrN_5_S, CAS No. 167393-62-6, Merck KGaA, Darmstadt, Germany), a colorimetric method of assessing cell survival and proliferation. The nanoparticles were diluted in RPMI medium with 10% fetal bovine serum (FBS) to concentrations of 5.0, 2.0, and 0.5 mg/mL. The effect was assessed after treatment intervals of 24, 48, and 72 h.

Cell proliferation was determined using the MTT assay as described by Mosmann [34]. The cells were incubated with sterile MTT solution (0.5 mg MTT in 10 mL RPMI), and 100 µL of the solution was added to each well, followed by incubation at 37 °C under 5% CO_2_. The stock solution was prepared at 500 mg/100 mL in PBS (Sigma-Aldrich, Darmstadt, Germany) and diluted 1:10 with RPMI medium containing 10% FBS before use. After 3 h, the MTT medium was removed and formazan crystals were dissolved in 100 µL of DMSO ((CH_3_)_2_SO, CAS No. 67-68-5, ACS reagent, ≥99.9%, Sigma-Aldrich, Darmstadt, Germany) + 96% ethanol (CH_3_CH_2_OH, CAS No. 64-17-5, 96%, EMPROVE^®^EXPERT, Ph. Eur., BP, ChP, Sigma-Aldrich, Gmbh, Wien, Austria) (1:1, *v*/*v*). Absorbance was measured at 540–620 nm on Citation 3 (Biotek, Agilent Technologies, Winooski, VT, USA).

The cytotoxic effects of the nanoformulations were assessed by measuring the percentage of cell viability compared to an untreated control group. To do this, the average absorbance of each group of cells was calculated and then compared to that of the control group. A lower absorbance indicates reduced cell viability, which reflects stronger cytotoxic effects.

### 2.3. In Vitro Cytocompatability Study in HaCaT Cells

HaCaT cells (CLS 300493), derived from immortalized human keratinocytes, serve as a model system to investigate the cytotoxic effects of LNPs and LCNPs. This cell line is available in the cell culture collection of the In Vitro Laboratory for Evaluation of Biological Activity and Toxicity at the Research and Development and Innovation Consortium. HaCaT cells are crucial in dermatological research, as they help elucidate the complex mechanisms of cell biology and pathology. This spontaneously immortalized cell line originates from the epidermal cells of an adult human and retains the ability to proliferate and differentiate, akin to basal keratinocytes in vivo. HaCaT cells provide a stable platform for studying epidermal differentiation and possess a high potential for proliferation.

HaCaT cells were cultured in T75 flasks using high-glucose DMEM (Sigma-Aldrich, Darmstadt, Germany) supplemented with 10% fetal bovine serum (FBS, Gibco, Waltham, MA, USA). The cells were incubated in a Panasonic HEPA Class 100 IncuSafe MCO incubator (Panasonic, Nijverheidsweg, The Netherlands) with humidified air containing 5% CO_2_ at a temperature of 37 °C. Before conducting experiments, the cells were trypsinized with a 0.25% trypsin-EDTA solution (sterile-filtered, BioReagent, suitable for cell culture, Sigma-Aldrich, Saint Louis, MA, USA) for 5 to 7 min. Following trypsinization, the cells were counted using a cell counter, stained with trypan blue, and diluted with DMEM containing 10% FBS. Non-tumor HaCaT cells (human epidermal keratinocyte line) were treated with nanoparticles in concentrations ranging from 0.5 mg/mL to 4 mg/mL and incubated for periods of 24, 48, and 72 h. Cell viability and proliferation were assessed using the MTT assay. The cultivation was carried out in 96-well plates, with an initial seeding density of 10,000 cells per well.

The data were exported and processed in Excel and GraphPad Prism 9.0. The preparation of the dilutions of the nanoparticles for treatment of the cells is presented in Table 2.

### 2.4. Antiradical Potential

Four spectrophotometric assays were used to study the antiradical potential: (a) DPPH (2,2-diphenyl,1-picrylhydrazyl); (b) ABTS^•+^ (2,2-azinobis(3-ethylbenzothiazoline-6-sulfonic acid)); (c) ferric reducing power (FRAP); and (d) nitric oxide (NO) scavenging activity was determined by Cuendet et al. [35], Adhikari et al. [36], Oyaizu [37], Shirwaikar et al. [38], respectively.

1,1-Diphenyl-2-picrylhydrazyl radical, 2,2-Diphenyl-1-(2,4,6-trinitrophenyl)hydrazyl (DPPH, C_18_H_12_N_5_O_6_, CAS No. 1898-66-4), 2,2-azinobis(3-ethylbenzothiazoline-6-sulfonic acid (ABTS™ chromophore, diammonium salt, C_18_H_18_N_4_O_6_S_4_·(NH_3_)_2_, CAS No: 30931-67-0), ethanol (EtOH, C_2_H_5_OH, p.a. ≥ 99.8%), sodium acetate (CH_3_COONa, CAS No.: 127-09-3, ACS reagent, ≥99.0%), Trolox (C_14_H_18_O_4_, CAS No.: 53188-07-1, 97%), ferric 2,4,6-tripyridyl-s-triazine (TPTZ, C_18_H_12_N_6_, CAS No.: 3682-35-7, for spectrophotometric det. (of Fe), ≥98%), FeCl3 (CAS No.: 7705-08-0, reagent grade, 97%), FeSO_4_.7H_2_O (CAS No.: 7782-63-0, ACS reagent, ≥99.0%) were supplied by Sigma-Aldrich (Saint Louis, MA, USA).

Direct antiradical potential was measured by spin-traps using EPR-X-band-BioSpin GmbH spectrometer e-scan (Bruker ER 116 DS, Ettlingen, Germany) methods.

#### 2.4.1. Lipid Peroxidation Inhibition

Lipid peroxidation inhibition was measured against phenyl N-tert-butylnitrone (PBN/DMSO solution; 5:0.04 mM) mixed with lignin, chitosan, and nanoparticles (1–3 mg/g), against the PBN/DMSO standard, at 23 °C [39].

#### 2.4.2. DPPH-H/R^•^ Capacity

The DPPH-H/R^•^ capacity was determined using the method of Dos Santos et al. [40]. DPPH-H/R^•^ (80 mM DPPH/98% EtOH) was mixed with lignin, chitosan, and nanoparticles (1–3 mg/g), at 5 min incubation at 23 °C. The capacity was calculated according to the equation:

(1) $$DPPH,\%=\frac{{(I}_{0}-I)}{I_{0}}\times 100,$$

where **I_0_** is the double integrated intensity of the DPPH signal for the blank sample and **I**— is the intensity of the double integrated DPPH signal of the test sample, measured after the addition of the DPPH-H/R^•^ scavenger.

#### 2.4.3. Superoxide Radicals BMPO/^•^O_2_^−^ Scavenging Potential

The spin trapping agent BMPO (PBS (phosphate-buffered saline, P-3813, Sigma-Aldrich, Saint Louis, MA, USA), pH = 7.4 and 50 μM DTPA (diethylenetriaminepentaacetic acid [(HOOCCH_2_)_2_NCH_2_CH_2_]_2_NCH_2_COOH, CAS No. 67-43-6, Sigma-Aldrich, Saint Louis, MA, USA) at a concentration of 100 mM in a mixture with hypoxanthine (1 mM/50 μL)/xanthine oxidase (XO, 1 U/mL, 50 μL) was used to generate BMPO/^•^O_2_^−^ radicals in lignin, chitosan, and the nanoparticles (1–3 mg/g) by stirring at 23 °C and in aerobic conditions. After 5 min incubation in a water bath at 40 °C, the samples were examined in triplicate, one scan per sample [41,42,43]. The BMPO/^•^O_2_^−^ scavenging potential was estimated by Equation (2):

(2) $$BMPO/•O_{2}^{-},\%=\frac{{(A}_{0}-A)}{A_{0}}\times 100,$$

where **A_0_** is the double integrated graph of the BMPO/^•^OOH adduct recorded in the control and **A**—the double integrated graph of the BMPO/^•^OOH spin adduct recorded on the tested sample.

#### 2.4.4. Hydroxyl Radicals BMPO/^•^OH Scavenging Potential

The generated hydroxyl radicals (^•^OH) were examined according to the methods of Wang et al. [43] and Zheleva et al. [44] with modifications. The mixture contained 20 mM BMPO (3,4-dihydro-2-methyl-1,1-dimethylethyl ester-2H-pyrrole-2-carboxylic acid-1-oxide, C_10_H_17_NO_3_, CAS No. 387334-31-8, Cayman Chemical, Ann Arbor, MI, USA), 0.2 mM FeSO_4_, 80 μL lignin, chitosan solutions, nanoparticles suspensions, and 2mM H_2_O_2_. Spectra were recorded 5 min after the reaction was started. The hydroxyl radicals BMPO/^•^OH scavenging potential was calculated by Equation (3):

(3) $$BMPO/•OH,\%=\frac{{(F}_{0}-F)}{F_{0}}\times 100,$$

where **F_0_** is the double integrated plot of the EPR spectrum of BMPO/•OH spin adduct registered in the control sample and **F**—is the double integrated plot of the EPR spectrum of BMPO spin adduct registered after addition of the tested sample.

All samples were tested in triplicate at the following constant working conditions: central field 3505–3516 G, microwave power 3.232–20.61 MW, modulation 5–10 G, a sweep width 100 G, a modulation amplitude—10 G, a sweep time—40.96 s, 1–5 scans per sample.

### 2.5. Antimicrobial Activity Testing

#### 2.5.1. Bacterial Strains

The bacterial strains included in the study were *Staphylococcus aureus* ATCC 25923, *Escherichia coli* ATCC 25922, *Pseudomonas aeruginosa* ATCC 27853, and the clinical isolate *Bacillus cereus*. Strains were kept at –80 °C in soyabean casein digest broth (TSB, HiMedia, Mumbai, India) supplemented with 20% *v*/*v* glycerol. Before experiments, strains were restored in tryptic soy agar (TSA, Sigma-Aldrich, Saint Louis, MA, USA) supplemented with 5% *v*/*v* defibrinated sheep blood (Tryptic Soy Blood Agar, Sigma-Aldrich, Saint Louis, MA, USA) and subcultured twice at 35 °C for 20–24 h.

#### 2.5.2. Antimicrobial Susceptibility Testing

The antimicrobial activity of the lignin and lignin+chitosan solutions and LNPs and LCNPs nanosuspensions against the tested strains was determined following the micro broth dilution method as described in [45]. Stock solutions of lignin and lignin+chitosan with an initial concentration of 36,000 mg/L and of LNPs and LCNPs nanosuspensions with an initial concentration of 36,400 mg/L were prepared. The antibacterial activity of the solutions was tested at two-fold dilutions prepared in cation-adjusted Mueller–Hinton broth (MHB, HiMedia, Mumbai, India) within the concentration range from 18,000 mg/L to 562.5 mg/L, and for the nanosuspensions—from 18,200 mg/L to 35.55 mg/L. The concentration ranges of the compounds were selected according to preliminary test results. The tests were performed in 96-well flat-bottom plates (Costar, Corning Incorporated, Kennebunk, ME, USA). Bacterial inocula from overnight cultures of strains on TSBA were prepared in physiological saline and brought to 0.5 McFarland standard (Densilameter II, Erba Lachema, Brno, Czech Republic). Plates were inoculated with the standardized inoculum diluted in MHB to achieve a final concentration of approximately 5 × 10^5^ cfu/mL. After sealing with gas permeable adhesive seals, the plates were aerobically incubated at 35 °C for 24 h. Positive controls of the growth (MHB and bacteria), sterility control (MHB with no bacteria) and controls of the solutions in their respective concentration ranges were also included. After incubation, the results were read at 620 nm wavelength on Microplate Photometer (BioSan, Riga, Latvia). Minimum inhibitory concentrations (MIC) were defined as the lowest concentrations of the tested compounds resulting in optical density (OD) value close to the negative controls (no growth). The assays were performed in triplicate with three independent experiments.

#### 2.5.3. Minimum Bactericidal Concentrations (MBCs)

The minimum bactericidal concentration (MBC), defined as the lowest concentration of solutions that kills 99.9% of bacteria, was determined for the solutions and nanosuspensions. The method was done by transferring the inoculum in the wells from the MIC test plate that showed no growth on Mueller–Hinton agar (MHA) plates. Agar plates were further incubated aerobically for 24 h at 35 °C. The lowest concentration of solutions/suspensions which exhibited no bacterial growth was considered the MBC. The experiments were performed in triplicate for each individual strain.

### 2.6. Statistical Analyses

All obtained results were subjected to statistical data processing using GraphPad Prizm 10.6 (GraphPad Software Inc., Boston, MA, USA) and XLSTAT version 2021.5 statistical software for Excel (Microsoft Corporation, Redmond, WA, USA). Numerical data were expressed as mean ± standard deviation (±SD) and presented graphically. Statistical significance within and between the treated groups and the untreated control was evaluated using one-way analysis of variance (ANOVA), followed by Dunnett’s multiple comparisons test or Tukey’s post-hoc test. Differences were considered statistically significant at *p* < 0.05. EPR spectral processing was accomplished using Bruker WIN-EPR SimFonia 1.2/6130860 software after double integration.

### 2.7. Ethical Statement

HaCaT (CLS 300493) and L929 (ATCC CCL-1) cell lines are available in the cell culture collection of the In Vitro Laboratory for Evaluation of Biological Activity and Toxicity at the Research and Development and Innovation Consortium, Sofia, Bulgaria.

## 3. Results and Discussion

### 3.1. Physicochemical, Morphological, and Spectral Characteristics of LNPs and LCNPs

The ζ-potential values for LNPs and LCNPs were experimentally determined in our previously published study as −30.13 ± 1.21 mV and −39.51 ± 1.98 mV, respectively [17]. Both mean values lie at or below the commonly cited ±30 mV threshold for colloidal stability, so both formulations would classically be categorized as anionic and likely to exhibit good electrostatic stabilization under the measurement conditions. Recent studies on lignin–chitosan nanoformulations have shown that anionic surfaces exhibit markedly reduced electrostatic interactions with negatively charged cellular membranes, which is associated with lower membrane-disruptive toxicity compared with cationic counterparts [33,46]. Additionally, lignin-based nanocarriers with negative ζ-potential have been reported to display reduced cytotoxicity due to weaker charge-mediated adhesion to cell membranes [22].

The more negative ζ-potential observed for LCNPs (−39.51 ± 1.64 mV) compared to LNPs (−30.13 ± 1.21 mV) (Table 1) can be attributed to the pH-dependent ionization behavior of both lignin and chitosan. In alkaline medium (pH 9.15–9.65), the -NH_2_ groups of chitosan (pK_a_ ≈ 6.3–6.5) are deprotonated, resulting in a loss of their typical cationic character. Consequently, chitosan does not electrostatically completely neutralize the negatively charged lignin surface. Instead, adsorption probably occurs through H-bonding and other non-electrostatic interactions, promoting surface rearrangement and enhanced exposure of the deprotonated phenolic and -COOH groups of lignin. The latter may lead to increased effective surface charge density. Furthermore, the formation of a thin polymer layer can shift the shear plane outward, contributing to a more negative measured electrokinetic potential. The increase in magnitude of the ζ-potential, together with the preserved particle size and morphology, suggests improved electrostatic stabilization rather than charge neutralization upon chitosan co-assembly.

The average size of LNPs was within the range from 50.6 ± 2.02 to 82.5 ± 4.12 nm, and the average size of LCNPs ranged from 56.3 ± 2.85 to 72.8 ± 3.94 nm [17]. The TEM analyses revealed that both LNPs and LCNPs exhibit homogeneous, spherical, and well-defined morphologies, with a dense arrangement indicative of polymeric network formation. Notably, smaller particle sizes (<100 nm) observed for both LNPs and LCNPs are favorable for enhanced cellular uptake and biodistribution, supporting potential applications in targeted delivery [17,33]. XRD analysis showed that both nanoparticle systems are predominantly amorphous, as reflected by broad diffraction halos. This poor crystallinity is attributed to the irregular three-dimensional polymeric structure of lignin and, in the case of LCNPs, the amorphous nature of the chitosan matrix [17,47]. FTIR spectroscopy provided insights into the probable chemical interactions within the nanoparticles. For LCNPs, shifts in the characteristic amide I and II bands of chitosan and -OH peaks of lignin indicated H-bonding between lignin and chitosan, reinforcing the structural stability of the composite nanoparticles. Similarly, LNPs exhibited spectral features consistent with aromatic, -OH, and -C=O groups [17].

In summary, while LNPs and LCNPs have comparable morphologies, size ranges, and amorphous characters, LCNPs benefit from additional hydrogen-bonding interactions provided by chitosan, potentially enhancing nanoparticle stability, bioadhesion, and interaction with biological membranes. LNPs, being purely lignin-based, provide a simpler system with slightly more size variability but maintain efficient encapsulation of bioactive compounds [17].

### 3.2. In Vitro Cytotoxicity Assessment

**Figure 1:**
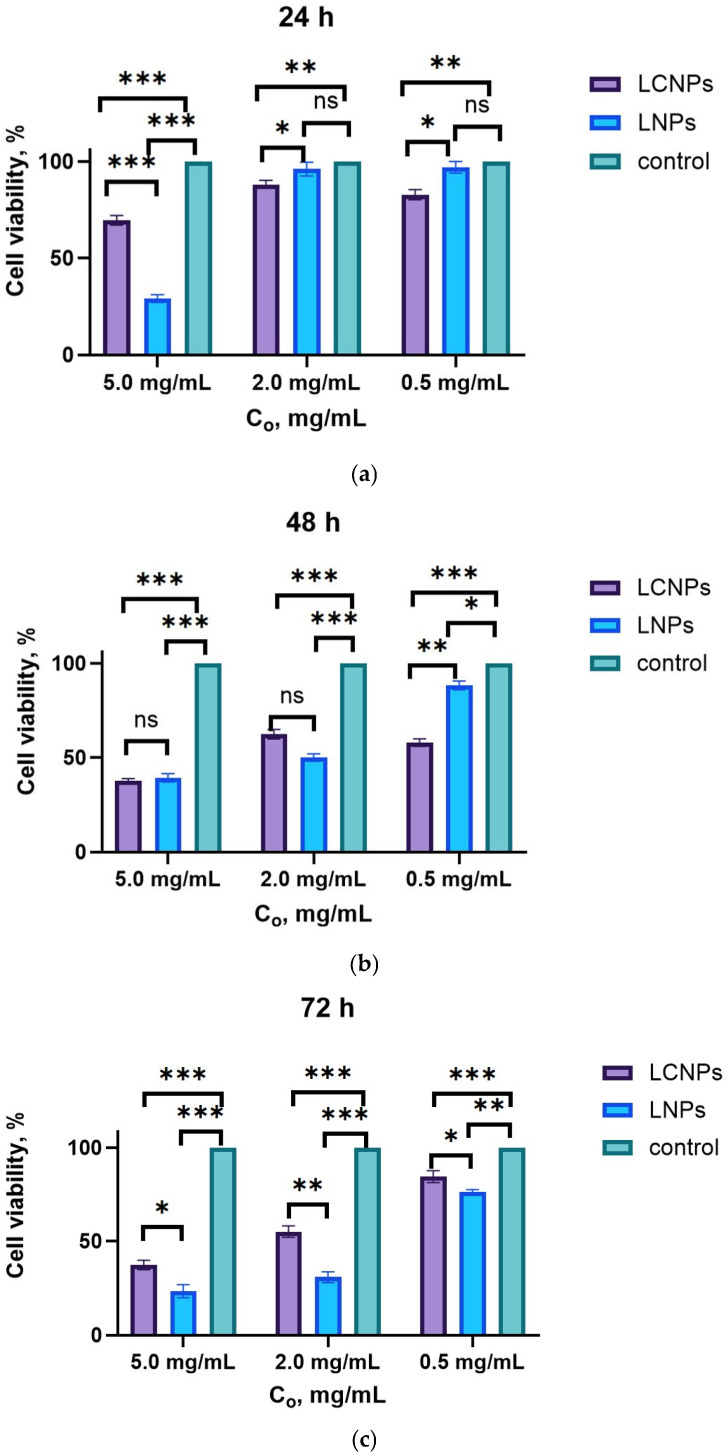
The effects of nanoparticles on the viability and proliferative activity of L929 fibroblast cells were examined under laboratory conditions. The cells were seeded at a density of 1 × 10^4^ cells per well in 96-well plates and cultured for (**a**) 24 h, (**b**) 48 h, and (**c**) 72 h. Cell viability was assessed using the MTT assay. Data are expressed as mean ± SD of three independent experiments. Statistical significance between the various experimental groups and the untreated control was determined using one-way analysis of variance (ANOVA), followed by Dunnett’s multiple comparisons test conducted with GraphPad Prism version 9.0. The results are expressed as a percentage of cell viability compared to the untreated control. Statistical significance is indicated as follows: * *p* < 0.05; ** *p* < 0.01; *** *p* < 0.001; ns—non-significant.

The cytotoxicity of LCNPs) and LNPs was assessed across three concentrations (5.0, 2.0, and 0.5 mg/mL) and three exposure periods (24, 48, and 72 h) (Figure 1). A clear dose- and time-dependent reduction in cell viability was observed for both nanoparticle types. However, notable differences emerged between the two formulations.

At 24 h, LCNPs exhibited moderate cytotoxicity only at the highest concentration, maintaining 69.97% cell viability, whereas LNPs induced a pronounced reduction to 29.51%. At 2.0 and 0.5 mg/mL, both nanoparticle types demonstrated minimal cytotoxicity, with LCNPs showing 88.68% and 83.90% viability and LNPs showing 95.45% and 97.08%, respectively (Figure 1a). These findings indicate that LCNPs are less cytotoxic than LNPs under acute high-dose conditions. After 48 h, increased cytotoxicity was evident for both nanoparticle systems (Figure 1b). At 5.0 mg/mL, LCNPs and LNPs produced comparable viability values (36.40% and 39.92%, respectively). At 2.0 mg/mL, LCNPs maintained higher viability (62.35%) than LNPs (50.41%), suggesting improved biocompatibility through chitosan modification. At the lowest concentration, however, LNPs (88.37%) were better tolerated than LCNPs (58.07%), indicating a differential cellular response at prolonged low-dose exposure. The long-term 72 h exposure further amplified cytotoxicity, particularly for LNPs (Figure 1c). Viability in the 5.0 mg/mL group decreased to 23.66% for LNPs compared to 37.73% for LCNPs. Similarly, at 2.0 mg/mL, LCNPs exhibited higher viability (55.70%) relative to LNPs (31.63%). At 0.5 mg/mL, both nanoparticles remained significantly biocompatible, with LCNPs displaying slightly higher viability (85.88%) compared to LNPs (76.00%).

Across all exposure periods, LCNPs demonstrated a comparatively more favorable cytotoxicity profile than LNPs, particularly at high and intermediate concentrations within the tested in vitro conditions. Chitosan functionalization appears to mitigate the cytotoxic effects associated with unmodified lignin nanoparticles, likely due to enhanced surface stability and reduced adverse cellular interactions. Both nanoparticle types exhibited time-dependent cytotoxicity. However, LCNPs maintained higher viability values under most conditions, supporting their potential advantage for biomedical applications requiring prolonged biocompatibility.

**Figure 2:**
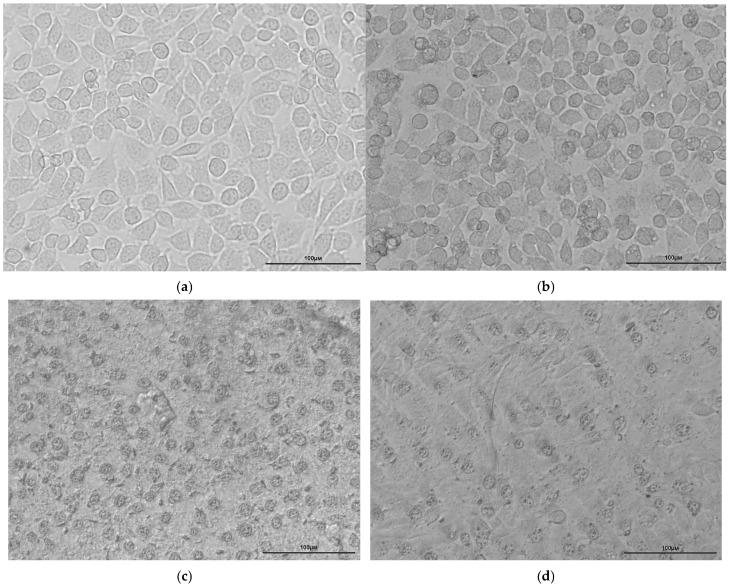
Light microscopic analysis performed 2 h after cell treatment. (**a**) Untreated cells—Characterized by regular spherical shape and clearly visible nuclei with intact nuclear membrane. The cytoplasm is uniformly stained, without signs of apoptosis or necrosis. Single cells in mitotic process are observed. (**b**) Cells treated with LCNPs 0.5 mg/mL—At some places, nuclei with normal morphology and nuclei in mitosis are observed. Enlarged cells and nuclei with altered nuclear structures are observed. (**c**) Cells treated with LCNPs 2.0 mg/mL—Partially under the layer of nanoparticles, cells with normal cell structure are observed, where the nuclei and cell membrane appear intact. The observed granular structures cannot be accurately characterized due to a layer of nanoparticles. (**d**) Cells treated with LCNPs 5.0 mg/mL—The shape and size of the cells are not visible. There are normal intact nuclear structures. Fragmented nuclei are also observed. Scale bar = 100 µm (applies to all panels).

Light microscopy analysis supported the quantitative cytotoxicity results (Figure 2). Untreated L929 fibroblasts displayed uniform morphology, intact nuclear membranes, and occasional mitotic figures (Figure 2a). Cells treated with 0.5 mg/mL LCNPs retained typical nuclear architecture, although some enlarged nuclei and subtle structural alterations were observed (Figure 2b). At 2.0 mg/mL, cellular boundaries and nuclear membranes generally remained intact, although visualization was partially obscured by nanoparticle deposition (Figure 2c). At the highest tested concentration (5.0 mg/mL), intact nuclear structures were still present, but occasional fragmented nuclei were observed, suggesting the onset of mild stress responses at elevated nanoparticle loads (Figure 2d).

The biocompatibility of LCNPs observed in this study is consistent with previous reports describing the low toxicity of lignin-based or chitosan-modified nanomaterials. Imiquimod-loaded nanostructured biofilms, for example, exhibited selective cytotoxicity toward melanoma cells (B16-F10) while exerting minimal effects on normal fibroblasts (L929) coupled with non-hemolytic behavior, underscoring their suitability for topical biomedical applications [7]. The study of Harper et al. on lignin nanoparticles (LNPs) and chitosan-coated LNPs (Ch-LNPs) in embryonic zebrafish demonstrated that unmodified LNPs exhibited minimal toxicity, whereas high concentrations of Ch-LNPs induced increased mortality and sublethal developmental endpoints. These findings highlight the importance of nanoparticle surface chemistry in modulating in vivo biocompatibility [24]. Additional evidence supporting the benign nature of lignin- and chitosan-based biomaterials is provided by a study on the cytotoxicity assessment of chitosan–alkali lignin xerogels performed using the MTT assay. The results obtained provided compelling evidence of its biocompatibility and suitability for biomedical applications. The gels were non-toxic to mesenchymal stem cells and remained non-toxic to zebrafish embryos at concentrations up to 100 µg/mL, indicating a broad safety margin across biological models. Moreover, the material provided a favorable microenvironment for cell adhesion and proliferation, reinforcing its applicability as a scaffold capable of supporting tissue regeneration. Notably, NIH 3T3 fibroblast cells demonstrated enhanced migratory behavior in the presence of the hydrogel, suggesting additional potential for wound-healing applications where guided cell migration is critical [30].

Overall, the results of the present study, together with supporting literature, indicate that LCNPs exhibit favorable cytocompatibility in the evaluated in vitro models. While minor structural alterations and nuclear fragmentation were observed at elevated nanoparticle levels, the general preservation of cell viability and morphology points to a favorable safety profile. Nevertheless, further mechanistic investigations, including assessments of oxidative stress, membrane interactions, and long-term cellular response, are warranted to fully elucidate nanoparticle–cell interactions and confirm the suitability of LCNPs for biomedical applications.

### 3.3. Cytocompatibility Assessment in HaCaT Cells

The metabolic activity of HaCaT cells following exposure to LNPs and LCNPs was assessed using the MTT assay over 24 h, 48 h, and 72 h periods (Table 3). Both nanoparticle types maintained relatively high cell viability at early time points, with a concentration- and time-dependent decrease observed over prolonged exposure. At 24 h, LNPs- and LCNPs-treated cells exhibited viabilities in the ranges of approximately 74–85% and 82–88%, respectively, demonstrating good initial cytocompatibility across the tested concentrations of 0.5, 1.0, 2.0 and 4.0 mg/mL. LCNPs consistently produced slightly higher viability than LNPs, suggesting that chitosan functionalization may improve cellular tolerance or mitigate acute stress. Even at the highest concentration tested (4.0 mg/mL), viability remained above 77% for LNPs and 82% for LCNPs, confirming minimal early cytotoxicity. After 48 h, cell viability began to decline. LNPs-treated cells showed a concentration-dependent reduction from approximately 82% at 0.5 mg/mL to 66% at 4.0 mg/mL. LCNP-treated cells exhibited a similar trend with a decrease from 80% at 0.5 mg/mL to 62% at 4.0 mg/mL. These results indicate the onset of time-dependent cytotoxic effects, potentially due to intracellular accumulation or interference with metabolic pathways. By 72 h, the differences between the two nanoparticle types became more pronounced: with a marked decline in the cell viability (31–68%) induced by LNPs compared to the substantially higher viability values (57–78%) maintained by LCNPs. The preserved metabolic activity in the cells treated with LCNPs suggests that chitosan co-assembly may mitigate cytotoxicity, possibly by reducing oxidative stress or modulating nanoparticle/cell interactions.

The values of the half-maximal inhibitory concentrations (IC_50_) for LNPs and LCNPs are summarized in Table 3. The high IC_50_ values registered at 24 h are associated with low acute cytotoxicity. LCNPs demonstrated the highest IC_50_ (12.26 mg/mL), consistent with the viability data showing enhanced early tolerance. At 48 h, IC_50_ values declined for both nanoformulations, indicating increasing cytotoxicity over time. After 72 h, LNPs produced the lowest IC_50_ (1.379 mg/mL) reflecting greater long-term cytotoxicity. In contrast, LCNPs maintained higher IC_50_ values (3.054 mg/mL), which is indicative of better cytocompatibility under prolonged exposure. Consequently, LCNPs demonstrate comparatively higher long-term cytocompatibility than unmodified LNPs within the tested in vitro conditions. Chitosan probably provides a protective effect, preserving metabolic activity and reducing cytotoxicity over extended periods. These features suggest that LCNPs may represent promising candidates for further investigation in regenerative biomaterial systems including wound-healing and sustained-release therapeutic platforms, where prolonged cell–nanomaterial interactions are critical.

The presented findings are consistent with literature scientific reports where chitosan–lignin hydrogels demonstrated biocompatibility while supporting keratinocyte proliferation, suggesting that surface functionalization can modulate oxidative stress and improve long-term cellular tolerance. The renewable nature and cost-effectiveness of the two-component conjugated biopolymer formulations further enhance their appeal for biomedical applications [30]. Previous in vivo studies using lignin–chitosan hydrogels support our in vitro findings, reporting accelerated wound healing (91% wound closure within 10 days, significantly surpassing the 61% closure observed in untreated controls) in rat models [27]. The enhanced wound repair potential was attributed to the antioxidant properties of both lignin- and chitosan-based on local cellular microenvironment modulation and promotion of tissue regeneration. The authors also reported 50% anticancer efficacy of the hydrogels against MCF-7 breast cancer cells, highlighting their multifunctional potential in biomedical applications [16].

The current results, together with prior literature data, suggest a possible basis for the wound-healing applicability of hybrid lignin–chitosan nanoformulations, although additional functional studies are required to confirm their regenerative performance. The preserved viability and sustained metabolic activity of keratinocytes in the presence of LCNPs suggest that these nanoparticles may be suitable candidates for incorporation into biomaterial scaffolds or hydrogels intended for skin-related tissue engineering applications.

It should be noted that the MTT assay primarily reflects cellular metabolic activity and therefore provides an indirect measure of cell viability rather than a comprehensive evaluation of biocompatibility or regenerative capacity. Consequently, while the higher viability values and IC_50_ parameters observed for LCNPs compared with LNPs suggest improved cytocompatibility within the tested in vitro models, these results should be interpreted as preliminary indicators rather than definitive proof of regenerative potential. The preservation of fibroblast and keratinocyte metabolic activity, together with the largely maintained cellular morphology observed by light microscopy, supports the hypothesis that chitosan-functionalized lignin nanoparticles may provide a favorable environment for skin-related cellular systems. Nevertheless, further investigations involving complementary biological assays, including cell migration, proliferation markers, oxidative stress evaluation, and in vivo wound-healing models, will be required to elucidate in detail the regenerative capabilities and long-term biocompatibility of LCNP-based biomaterials.

### 3.4. In Vitro Radical-Scavenging Potential

EPR spectroscopy is among the very few methods that acknowledge direct detection of radicals in complex systems such as natural mineral composites, biopolymer macromolecules, intact cells, and tissue samples. Thus, it has been highlighted as the “gold standard” for ROS detection and characterization in various chemical and biological systems. Quantitative free radical scavenging capacity toward the stable free radical 1,1-diphenyl-2-picryl hydrazyl (DPPH) can be investigated by electron paramagnetic resonance (EPR) spectroscopy. In addition, various spin traps have been developed that aid the detection of specific types of radicals or radical formation in particular compartments. The nitrone type spin trap N-tert-butyl-α-phenylnitrone (PBN) typically produces wider spectrum of long-lived adducts. As the radical is remote from the nitroxide group, the identification of the added species is dependent basically on the effect of this species on the hyperfine couplings arising from the magnetic nuclei present in the spin trap. The more novel spin trap 5-tert-butoxycarbonyl-5-methyl-1-pyrroline-N-oxide (BMPO) traps ^•^O_2_^−^ and therefore produces the relatively stable ^•^BMPO-OOH spin adduct, which facilitates detection in complex systems [40].

**Figure 3:**
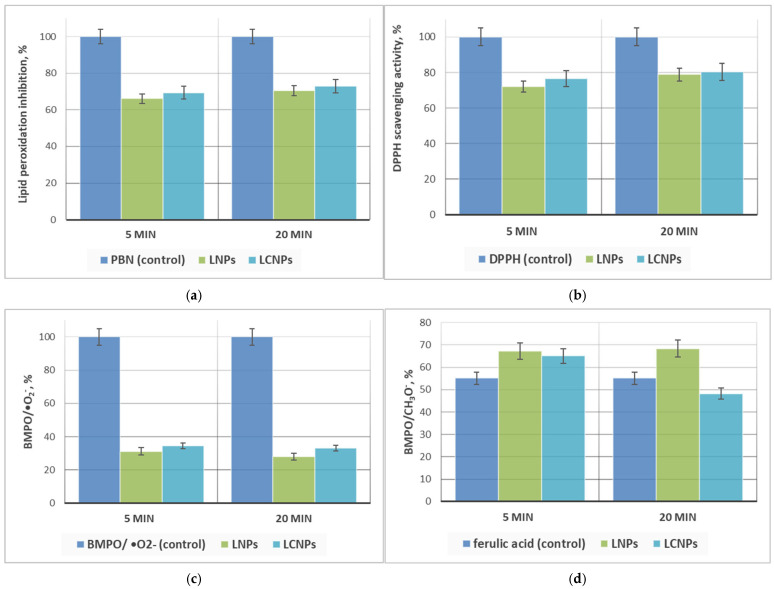
EPR (**a**) lipid peroxidation, (**b**) DPPH, (**c**) BMPO/^•^O_2_^−^ and (**d**) BMPO/CH_3_O^•^ radical scavenging capacity of LNPs and LCNPs.

EPR spectroscopy was employed to evaluate the radical-scavenging potential of LNPs and LCNPs against lipid peroxidation, DPPH radicals, superoxide (^•^O_2_^−^), and methoxy radicals (CH_3_O^•^) (Figure 3) [41,48]. Both nanoformulations demonstrated substantial inhibition of lipid peroxidation (at 5 min: 66.1% and 69.3%, respectively; at 20 min: 70.5% and 72.8%, respectively) compared to the spin trap PBN (100%). The slightly higher activity of LCNPs suggests that chitosan incorporation enhances interaction with lipid radicals, possibly via electrostatic stabilization or improved dispersion in the lipid environment. With respect to DPPH radical scavenging, LNPs exhibited 72.1% and 78.9% inhibition at 5 and 20 min, respectively, while LCNPs displayed 76.5% and 80.4% activity. These results confirm that both nanoparticle types possess strong hydrogen- or electron-donating capacity, with LCNPs slightly superior at early time points, probably due to synergistic effects between the conjugated biopolymers lignin and chitosan. According to modern scientific literature, the DPPH assay is influenced by various molecular structural characteristics, and the radical scavenging potential of polyphenolic compounds depends on the number and position of aliphatic/aromatic -OH groups as well as the presence of other substituents and functional groups [49,50]. In this respect, the increased number of phenolic -OH, -OCH_3_ groups at the aromatic rings and the double C=C bonds between the outermost carbon atoms in the side chains of the lignin macromolecules positively influence its DPPH potential and substantiate the highest registered DPPH scavenging potential of the heterobiopolymer-based nanoformulations [23].

BMPO was used in the present study as a spin-trapping agent to scavenge superoxide (^•^O_2_^−^) and methoxy (CH_3_O^•^) free radicals via EPR spectroscopy. Regarding superoxide radical (BMPO/^•^O_2_^−^) scavenging activity, a higher inhibition potential was registered for LCNPs. The latter can be attributed to the physicochemical contribution of chitosan. The protonated amino groups (-NH_3_^+^) of chitosan confer a positively charged, hydrophilic surface that promotes electrostatic attraction of the negatively charged superoxide radical, thereby increasing its local concentration at the nanoparticle interface and facilitating electron transfer reactions. In addition, chitosan contains primary amino groups (-NH_2_/-NH_3_^+^) and hydroxyl groups (-OH) that can participate in radical-quenching reactions. The amino groups can donate electrons or H-atoms to radicals, forming relatively stable amino-centered species, while the -OH groups can provide supplementary H-donating capacity. These functionalities act synergistically with the phenolic moieties of lignin, further enhancing ^•^O_2_^−^ scavenging. In contrast, the lower methoxy radical BMPO/CH_3_O^•^ scavenging activity of LCNPs relative to LNPs could be attributed to partial shielding of lignin phenolic and aromatic sites by the co-assembled chitosan macromolecules, reducing the availability of the functional groups most reactive toward neutral alkoxy radicals. The more phenolic-rich surface of LNPs thus enables more efficient interactions with CH_3_O^•^ radicals, leading to superior scavenging compared to the chitosan-modified nanoparticles.

The Tukey’s post-hoc multiple comparison analysis following one-way ANOVA for the experimentally obtained data of the lipid peroxidation, DPPH, and BMPO-based radical scavenging potentials of both types of nanoparticles proved their statistical significance (Table 4).

**Figure 4:**
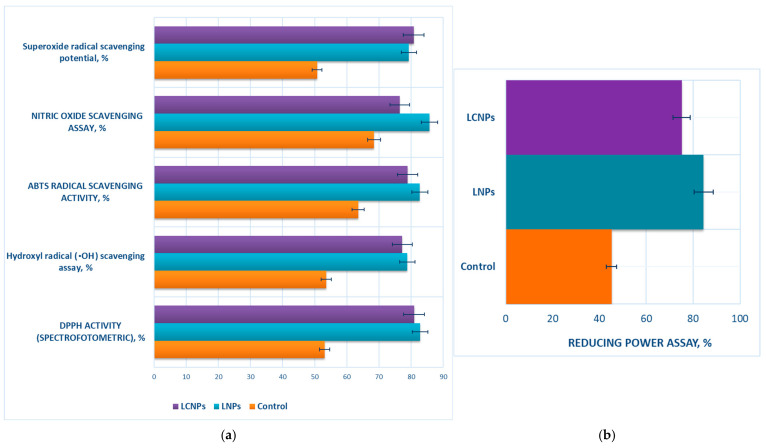
Spectrophotometrically determined (**a**) DPPH, hydroxyl radical, ABTS, nitric oxide, superoxide radical scavenging activity and (**b**) reducing power of LNPs and LCNPs.

Spectroscopic assays further quantified the antioxidant potential of the nanoparticles across multiple radical systems and reducing capacities (Figure 4). LNPs exhibited significantly higher ferric reducing power (FRAP) activity than LCNPs and Trolox, indicating superior electron-donating potential attributable to the unmodified phenolic hydroxyl groups of the nanoformulation. Although both nanoparticles demonstrated strong DPPH (82.8% for LNPs, 80.9% for LCNPs) and ABTS scavenging activities (82.7% for LNPs, 78.9% for LCNPs) surpassing the Trolox standard, the lignin nanoformulation consistently exhibited higher potential, suggesting that chitosan incorporation slightly diminishes phenolic-mediated radical neutralization. A similar trend was observed with respect to hydroxyl (^•^OH) and nitric oxide (NO) radical scavenging activity, with LNPs outperforming LNCPs. These results confirm the assumption that lignin phenolic groups dominate over the neutralization of highly reactive radicals, while chitosan probably partially shields these functional groups.

The Tukey’s post-hoc multiple comparison analysis following one-way ANOVA for the experimentally obtained data of the FRAP, DPPH, hydroxyl radical, ABTS, nitric oxide, and superoxide radical scavenging activity of LNPs and LCNPs is presented in Table S2.

Among both the EPR and spectroscopic analyses, LNPs consistently exhibit stronger intrinsic antioxidant activity, particularly for radicals dependent on phenolic hydrogen donation (^•^OH, NO, ABTS), while LCNPs display selective advantages, such as modestly improved lipid peroxidation and superoxide radical scavenging, likely due to chitosan-mediated stabilization and enhanced dispensability of the nanoformulations. However, CH_3_O^•^ radical scavenging and FRAP are reduced by LCNPs, indicating that chitosan incorporation may sterically hinder or shield the active phenolic sites of lignin. The differences observed between the two nanoparticle systems highlight the fact that the antioxidant mechanism is highly radical-specific. In this respect, the phenolic hydroxyls in lignin are the probable primary mediators for highly reactive radicals, while chitosan contributes to solubility and electrostatic stabilization, particularly in lipid and superoxide systems.

The notable antioxidant potential observed for LNPs and LCNPs in the present study is consistent with previous reports on lignin–chitosan nanocomposites and hydrogels. According to the study of Jassal et al., lignin serves as a natural capping, reducing, and stabilizing template due to its aromatic structure and multifunctional -OH, -OCH_3_, and -COOH groups, which facilitate H-donation and electron transfer to neutralize reactive radicals. Chitosan further contributes to the overall antioxidant activity through its free -NH_2_ and -OH groups, which interact with ROS to form stable macromolecular radicals and can subsequently accept H^+^, producing ammonium (-NH_3_^+^) groups that stabilize the radical species [7]. An assumption that incorporation of chitosan into lignin nanoparticles enhanced dispensability and surface interactions, which could lead to improved radical scavenging in specific systems such as lipid peroxidation and superoxide radicals, was substantiated by the observations of Xu et al. [51]. They reported that H-bonding and cross-linking facilitated the formation of stable nanocomposites with high antioxidant efficiency, as measured by DPPH assay for lignin–chitosan hydrogel polymers used to immobilize gold nanoparticles. Previous studies also highlighted the dose-dependent nature of the antioxidant activity for lignin–chitosan films, where higher concentrations enhanced radical scavenging [7]. The FRAP and DPPH scavenging analyses of Abdullah et al. corroborated these findings, demonstrating that lignin–chitosan nanocomposites possess a high capacity to donate electrons and neutralize free radicals, with reported DPPH activity up to 89% [16]. These observations align with prior reports on lignin/chitosan/Au NPs, where chitosan enhanced the stabilization of reactive species while lignin remained the primary contributor to electron transfer and radical neutralization [31].

Chitosan incorporation enhances radical-specific antioxidant activity in LCNPs, particularly against lipid peroxyl and superoxide radicals, likely due to its amino and hydroxyl functionalities, which complement lignin-mediated phenolic radical scavenging. This suggests potential for mitigating oxidative stress, although further in vitro and in vivo studies are needed to confirm its effects in biological systems.

In conclusion, these findings indicate that the co-presence of lignin and chitosan in nanoparticle formulations modulate antioxidant activity in a radical-specific manner as lignin drives phenolic-mediated radical scavenging, while chitosan enhances surface interactions and stabilization of reactive intermediates. This co-action not only explains the potent antioxidant activity observed in the current study but also underscores the potential of lignin–chitosan hybrid nanomaterials for biomedical applications, including ROS mitigation, oxidative stress reduction, and enhanced healing processes.

### 3.5. Antimicrobial Activity

The present study investigated the minimum inhibitory concentration (MIC) and minimum bactericidal concentration (MBC) of LNP and LCNPs against selected Gram-positive and Gram-negative bacteria. The determination of MBC is crucial to assessing the efficacy of antimicrobial agents. Unlike MIC, which indicates growth inhibition, MBC determines the lowest concentration required to kill 99.9% of bacterial cells. The MIC and MBC were determined to be in the concentration range of 18,200 mg/L to 35.55 mg/L final concentration of the nanoparticles suspensions and from 18,000 mg/L to 2250 mg/L of the lignin and lignin+chitosan solutions against the four bacterial strains. The results obtained are presented in Table 5.

Across all tested formulations, three overarching trends were consistently observed. First, solutions of lignin and lignin/chitosan mixtures exhibited weak inhibitory activity, with MICs in the 10^3^–10^4^ mg/L range or undetectable inhibitory effects at the highest tested concentrations. Second, the conversion of alkali lignin into lignin nanoparticles resulted in 8- to 13-fold reductions in MIC values, indicating a profound enhancement in apparent antimicrobial potency. Third, hybrid nanoparticles, generated by the co-assembly of lignin with chitosan, exhibited the greatest antimicrobial activity across all four reference bacterial strains, with the most pronounced relative improvement recorded for *S. aureus* vs. that of LNPs. Collectively, these results establish nanoscale formulation as the principal determinant of enhanced antimicrobial performance and further demonstrate that compositional tuning via chitosan incorporation provides an additional, mechanistically distinct mode of potentiation beyond the effects of particle size.

The substantial decrease in MIC values observed upon transitioning from soluble lignin to LNPs is consistent with recently reported findings attributing enhanced antimicrobial activity to nanostructured lignin [29]. Increased specific surface area, local density of phenolic groups, and improved contact delivery to the bacterial envelope have been identified as major contributors to this effect [52]. In agreement with these reports and comprehensive reviews, phenolated or surface-modified lignin nanoparticles can function as direct antibacterial agents or serve as potent carriers that enhance the activity of co-formulated antimicrobial compounds [53]. From a mechanistic perspective, nanoparticle formation concentrates the redox-active and amphipathic moieties of lignin at the particle-cell interface, increasing the likelihood of membrane perturbation, adsorption, and localized oxidative stress [54]. These physicochemical properties align with the order-of-magnitude reductions in MIC observed for both the LNP and LCNP suspensions (Table 5).

Chitosan introduces a complementary mechanism of action. Protonated amine groups (-NH_3_^+^) confer a positive surface charge that promotes electrostatic attraction to negatively charged bacterial surfaces—lipopolysaccharides in Gram-negative strains and teichoic acids in Gram-positive bacteria. The intrinsic antimicrobial properties of chitosan—owing to membrane permeability, metal-ion chelation, and interference with nutrient uptake—are well established [28]. These attributes rationally explain the consistently equal or improved MIC values observed for LCNPs relative to LNPs, most likely due to enhanced nanoparticle–cell association facilitated by the cationic surface of chitosan.

The greatest relative enhancement in susceptibility was observed for *B. cereus*, for which LCNPs MIC values decreased approximately two-fold relative to LNPs. Gram-positive bacteria, characterized by thick peptidoglycan layers and anionic teichoic acids, are particularly susceptible to cationic carriers such as chitosan, which promotes efficient surface capture and amplifies the effects of membrane-active phenolic moieties. Although Gram-negative bacteria (*E. coli*, *P. aeruginosa*) possess an outer membrane that restricts nanoparticle penetration, both LNPs and LCNPs exhibited improved MIC values relative to soluble lignin, indicating that nanoscale formulation enhances effective delivery to the outer membrane sufficiently to induce bacteriostatic effects. These strain-dependent trends mirror previous comparative studies of LNPs and lignin-based hybrid nanomaterials [29,52].

MBC assessments revealed limited bactericidal activity within the examined concentration range (Table 5). For most strain-formulation combinations, MBC values were not achieved at the highest tested concentrations, with *B. cereus* showing the only consistent exception. Thus, under the conditions of this study, the nanoparticle formulations function mainly as bacteriostatic agents. This observation has important implications based on the assumption that materials that inhibit growth effectively but do not induce bactericidal effects at low concentrations are more appropriate for prophylactic or surface-protective applications (e.g., antimicrobial packaging, coatings, or antifouling surfaces) than for systemic therapeutic deployment. The broader literature likewise emphasizes the primary utility of lignin-based nanoparticles in preventive or combination antimicrobial strategies [29,52].

The present findings demonstrate that lignin is a sustainable and chemically versatile scaffold, in which antimicrobial performance is enhanced by nanoscale formulation and incorporation of cationic polymers. In this respect, the study of Khan et al. reported that lignosulfonate–chitosan nanoparticles with diameters of approximately 125 nm exhibited strong antibacterial activity against *S. aureus* and *E. coli*, high antioxidant performance, and UV-protective properties attributable to their polyphenolic content [12]. Similarly, PVA–chitosan/lignin@CdZnO hydrogels displayed synergistic antibacterial activity driven by reactive oxygen species generation, the release of Zn^2+^ and Cd^2+^ ions, and contributions from chitosan amino groups and lignin phenolic moieties, outperforming both CdZnO nanoparticles and lignin@CdZnO nanocomposites alone [16].

The MIC values of LNPs and LCNPs observed in this study are consistent with the previously reported antimicrobial activities of technical lignins and lignin-derived nanomaterials. The published data indicate that unmodified kraft, soda or organosolv lignins typically exhibit MIC values between 1 and 50 mg/mL, depending on the lignin source, molecular weight distribution, and bacterial strain [29,54]. Similar to our findings, Gram-positive bacteria are generally more susceptible than Gram-negative strains, which is attributed to differences in cell wall architecture and the presence of the outer membrane in Gram-negative organisms [55]. Nanostructuring of lignin has been shown to enhance antimicrobial activity through increased surface area and improved interaction with bacterial membranes [56].

Nevertheless, despite the observed enhancement, the MIC values remain substantially higher than those reported for conventional antibiotics. Clinically used antibiotics typically exhibit MIC values in the µg/mL range. For example, susceptibility breakpoints defined by the European Committee on Antimicrobial Susceptibility Testing (EUCAST) and the Clinical & Laboratory Standards Institute (CLSI) indicate MIC values of ≤2 µg/mL for vancomycin against *S. aureus*, ≤1 µg/mL for ceftriaxone against *E. coli*, and ≤2–8 µg/mL for meropenem against *P. aeruginosa* [57,58]. Therefore, although LNPs and LCNPs demonstrate in vitro antibacterial activity, their MIC and MBC values preclude systemic clinical application as therapeutic antibiotics.

Despite its limited clinical relevance for systemic antimicrobial therapy, this demonstrated antibacterial activity may still be valuable for non-systemic applications. High local concentrations can be tolerated in surface coatings, wound dressings, food packaging materials, and biomedical device coatings, where direct contact with microorganisms occurs without systemic absorption. In such contexts, lignin–chitosan nanoparticles may provide eco-friendly, bio-based antimicrobial functionality with a reduced risk of resistance development.

Overall, the present results substantiate the consensus that nanoscale formulation is critical for realizing lignin antimicrobial potential and chitosan incorporation further enhances potency, particularly against Gram-positive bacteria. The predominance of bacteriostatic effects and the relatively high mass concentrations required for inhibition underscore the need for caution when considering therapeutic applications. Continued optimization of nanoparticle size, surface chemistry, and co-formulation strategies will be essential for advancing lignin–chitosan nanomaterials toward practical, high-performance antimicrobial technologies.

### 3.6. Nanoarchitechture vs. Comparative Mechanistic Basis of the Reported In Vitro Biofunctionalities of LNPs and LCNPs

The present results demonstrate that the nanoarchitectural integration of lignin and chitosan into LCNPs generates a hybrid system in which biological performance is dictated by interfacial chemistry, surface charge distribution, and supramolecular organization. Compared with pure LNPs, the conjugated LCNPs exhibit a more favorable balance between redox activity and cytocompatibility, confirming that controlled biopolymer coupling can modulate both physicochemical and biofunctional outcomes.

**Scheme 1:**
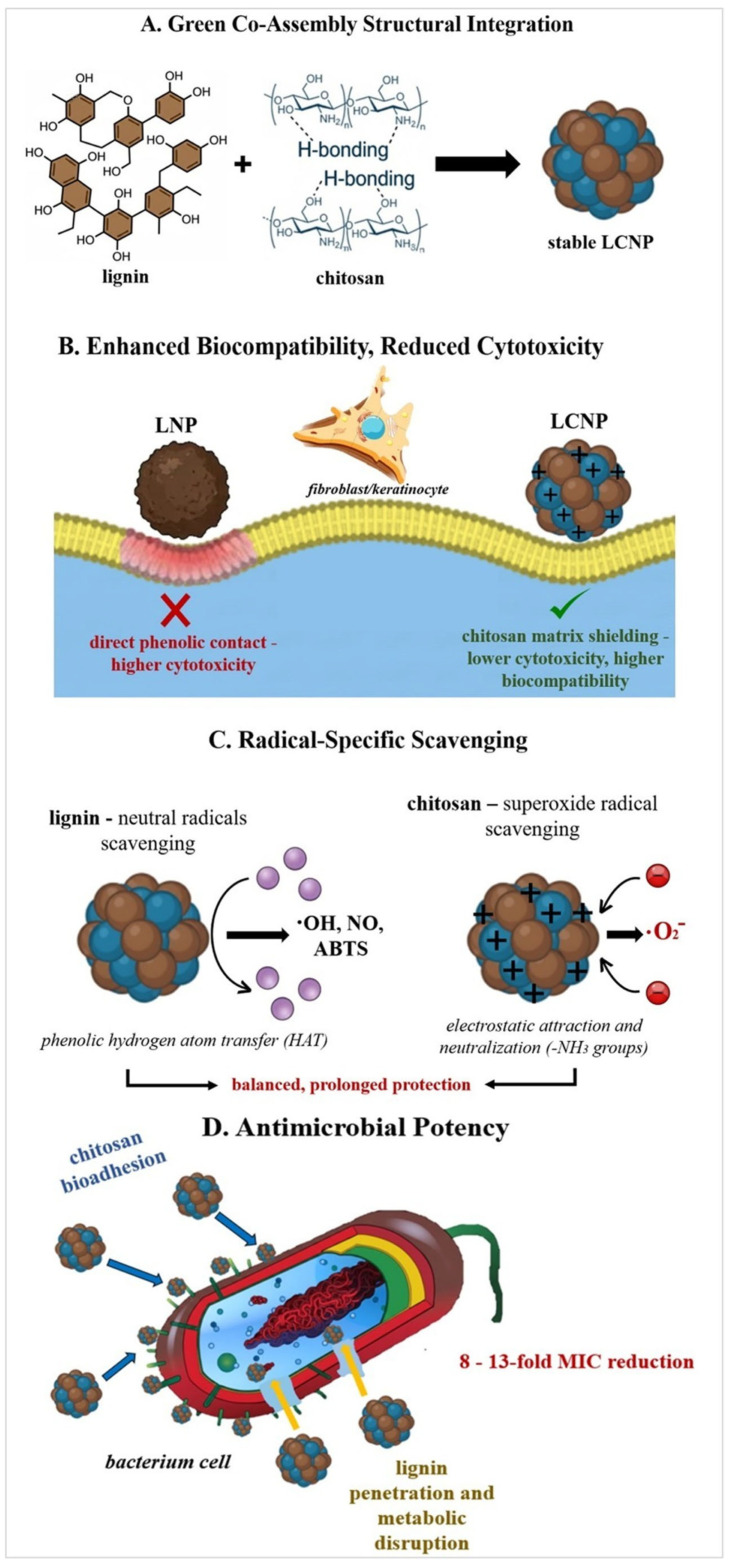
Schematic representation of the probable mechanistic basis of the reported in vitro biofunctionalities of the studied LNPs and LCNPs (Adapted from: [6,11,16,28,29,52,53,54,59]).

Cytocompatibility and Surface Shielding Effects: The comparative cytotoxicity analysis in L929 and HaCaT cells revealed a more pronounced safety profile for LCNPs relative to LNPs. This observation can be mechanistically explained by the polyglycan shielding effect provided by chitosan. LNPs expose a high density of phenolic hydroxyl and methoxy groups at the surface [33]. At elevated concentrations, these redox-active moieties may promote localized oxidative stress or membrane lipid peroxidation due to direct hydrophobic interactions with the lipid bilayer. In contrast, co-assembly with chitosan establishes extensive intermolecular H-bonding between lignin -OH groups and chitosan -NH_2_ groups, as confirmed by FTIR band shifts (amide I and II displacement) [17,32,33]. This interaction partially “masks” the reactive lignin surface, leading to: (i) a reduction of cell membrane disruption due to the hydrophilic chitosan’s prevention of the aggressive hydrophobic interaction of lignin with the cellular lipid bilayer; (ii) electrostatic stabilization due to the preventive action of the chitosan’s cationic nature against nanoparticle agglomeration in biological media, as noted by Yaneva et al. [52], ensuring a more uniform and less toxic cellular exposure (Scheme 1).

Additionally, potentiometric titration revealed a higher concentration of protonated -NH_3_^+^ groups (18–18.7 meq/g) compared to acidic sites (14–15 meq/g), confirming the dominance of surface -NH_3_^+^ functionalities under mildly acidic conditions [17,33]. These positively charged groups contribute to electrostatic stabilization in biological media, limiting nanoparticle aggregation and ensuring more homogeneous cellular exposure. This colloidal stability is consistent with the measured ζ-potential values, which fall beyond the ±30 mV threshold for stable dispersions, indicating robust electrostatic repulsion (Table 1).

Surface Charge, Stability, and Cellular Interaction: The ζ-potential analysis classified both LCNPs and LNPs as anionic systems, with the conjugated nanoformulation displaying a more pronounced negative value. The anionic character arises from the deprotonated phenolic and carboxyl groups of lignin at physiological pH, despite the presence of protonated chitosan amines. This balanced surface chemistry likely reduces nonspecific membrane disruption, as highly cationic systems are typically associated with increased cytotoxicity due to strong electrostatic attraction to negatively charged cellular membranes. TEM imaging confirmed homogeneous spherical morphology with nanoscale dimensions, while XRD analysis indicated predominantly amorphous structures [17,32,33,47]. This amorphous nature enhances the surface accessibility of functional groups and facilitates molecular diffusion within the matrix.

Antioxidant Mechanisms—Radical-Specific Activity: EPR and spectroscopic data demonstrate that antioxidant performance is radical-specific and mechanism-dependent. Pure LNPs predominantly operate through hydrogen atom transfer (HAT) and single electron transfer (SET) mechanisms driven by phenolic moieties, explaining their superior FRAP values and ^•^OH scavenging capacity. Conversely, LCNPs exhibit enhanced scavenging of superoxide radicals (^•^O_2_**^−^**), suggesting a charge-mediated capture mechanism. Electrostatic attraction between anionic superoxide species and surface -NH_3_^+^ groups likely increases radical proximity to the nanoparticle interface, where underlying lignin phenolics neutralize reactive species. This proximity-enhanced quenching represents a synergistic redox interface, combining electrostatic sequestration with chemical radical neutralization. Such cooperative behavior confirms that chitosan does not merely dilute lignin reactivity but also spatially reorganizes it for selective antioxidant optimization. This addition effect confirms the observations of Xie et al. regarding the role of chitosan in stabilizing reactive intermediates [31].

Antimicrobial Synergy and Multimodal Action: The significant reduction in MIC values upon nanoformulation suggests a transition from simple chemical inhibition to a multimodal mechanical–chemical attack on the bacterial envelope. For LCNPs, the enhanced antimicrobial potency against *S. aureus* and *B. cereus* is driven by a two-step mechanism. During the first stage of electrostatic sequestration, the -NH_3_^+^ groups of chitosan bind irreversibly to the teichoic acids in the membrane of the Gram-positive bacteria or to the lipopolysaccharides of the Gram-negative bacterial cell wall. The second stage of membrane permeabilization and oxidative stress induction comprises inducing structural damage to the cell membrane through hydrophobic insertion by the concentrated lignin core and the generation of localized reactive species, leading to cytoplasmic leakage and cell death. As supported by Reyes et al. (2024) and Wu et al. (2024), this dual mechanism prevents the bacteria from easily developing resistance, as the attack is both physical (surface charge) and chemical (redox-active) [28,29]. The high IC_50_ values and preserved metabolic activity in HaCaT cells propose that LCNPs act as bioactive scaffolds.

The ability to maintain high cell viability even at 72 h (up to 78% for LCNPs at 0.5 mg/mL) suggests that the hybrid nanoparticles modulate the local microenvironment by scavenging excess ROS, which is a primary inhibitor of the proliferative phase in wound healing. This mechanistic insight is supported by the findings of Dahlan et al. (2026), where the antioxidant properties of the lignin–chitosan complex promoted tissue regeneration [59]. The LCNPs developed here could serve not only as passive carriers but also as active participants in cellular redox homeostasis, making them ideal candidates for advanced dermatological applications.

### 3.7. Limitations of the Study

Despite the promising multifunctional performance of the newly synthesized lignin–chitosan hybrid nanoparticles (LCNPs), several limitations should be acknowledged.

The biocompatibility evaluation was restricted to in vitro models (L929 fibroblasts, HaCaT keratinocytes, and selected bacterial strains). While these systems are widely accepted for preliminary biocompatibility and antimicrobial screening, they cannot fully replicate the complexity of in vivo tissue microenvironments, immune responses, enzymatic degradation, and nanoparticle biodistribution. Therefore, the cytocompatibility and wound-healing potential of LCNPs require confirmation in relevant animal models before translational conclusions can be drawn. Furthermore, although LCNPs demonstrated preserved metabolic activity in HaCaT cells up to 72 h, longer exposure periods and more comprehensive toxicological profiling (e.g., genotoxicity, inflammatory markers, mitochondrial function, and oxidative DNA damage) have to be studied.

Such investigations are essential to confirm the long-term safety of these hybrid nanomaterials for dermatological or wound-healing applications. The MTT assay primarily reflects metabolic activity and does not directly assess cell proliferation, migration, or tissue regeneration; therefore, further mechanistic and functional studies are required to validate the regenerative performance of LCNP-based biomaterials.

Antimicrobial activity was assessed against a limited panel of bacterial strains. Although the observed reduction in MIC values indicates enhanced potency, broader-spectrum studies including clinically isolated, multidrug-resistant strains as well as biofilm-forming models are necessary to substantiate the proposed resistance-preventive dual mechanism. The long-term biodegradation kinetics and potential byproduct formation of LCNPs in biological environments were not systematically investigated. Given that lignin is structurally heterogeneous and chitosan properties depend on the degree of deacetylation and molecular weight, batch-to-batch variability may influence reproducibility and bioactivity.

In summary, while the present findings establish LCNPs as a promising bioactive nano-platform with synergistic antioxidant and antimicrobial properties, further in vivo validation, mechanistic refinement, extended microbiological assessment, and long-term safety studies are required to fully substantiate their clinical and translational potential.

## 4. Conclusions

The present study demonstrates that both LNPs and LCNPs possess favorable nanoscale characteristics, including spherical morphology, small particle size, negative surface charge, and amorphous structure, supporting their potential for biomedical delivery. Chitosan incorporation into LCNPs enhanced H-bonding interactions, providing improved structural stability, biocompatibility, and cellular tolerance compared with unmodified LNPs. LCNPs maintained higher cell viability and metabolic activity across prolonged exposure periods, confirming their suitability for regenerative medicine and wound-healing applications. Antioxidant analyses revealed that while LNPs exerted stronger phenolic-mediated radical scavenging, LCNPs exhibited selective improvements in lipid peroxidation and superoxide radical neutralization, highlighting the complementary roles of lignin and chitosan. Antimicrobial evaluations confirmed that nanoscale formulation substantially increased the bacteriostatic activity, with LCNPs demonstrating higher efficacy, particularly against Gram-positive bacteria, due to chitosan-mediated electrostatic interactions. Overall, the results indicate that lignin–chitosan hybrid nanoparticles combine biocompatibility, antioxidant potential, and antimicrobial activity, offering a multifunctional platform for biomedical applications such as tissue regeneration, oxidative stress mitigation, and surface-protective antimicrobial strategies. Furthermore, the established hierarchy of biological activity among the studied nanoparticles offers a foundation for optimizing their design in future in vitro and in vivo studies aimed at regenerative medicine, dermatology, and the development of biocompatible nanomaterials. Taken together, these observations underscore the potential of lignin–chitosan nanoparticle systems as advanced platforms for tissue engineering and wound healing applications. Future studies should explore in vivo efficacy, long-term safety, and mechanistic insights to fully realize the therapeutic potential of the investigated hybrid nanomaterials.

## 5. Patents

GB Patent Application No: GB2415240.7, Nanoparticle Compositions, Applicant: Trakia University, Stara Zagora, Bulgaria, 2024.

## Figures and Tables

**Table 1:** ξ-Potential, particle size, form, and pH of LNPs and LCNPs.

Nanoparticle Type	Size, nm	ξ-Potential, mV	Particles Form	pH	Ref.
LNPs	50.6 ± 2.02–82.5 ± 4.12	−30.13 ± 1.21	homogeneous surface, spherical, regular form	9.65	[17]
LCNPs	56.3 ± 1.83–72.8 ± 3.57	−39.51 ± 1.64	homogeneous surface, spherical, regular form	9.15	[33]

**Table 2:** Sample dilution and preparation for the in vitro regenerative essay.

Working Concentration, mg/mL	Volume of Initial Solution (C_0_ = 5 mg/mL)	DMEM + 10% FBS	Dilution Ratio
4	0.8 mL	0.2 mL	4:5
2	0.4 mL	0.6 mL	2:5
1	0.2 mL	0.8 mL	1:5
0.5	0.1 mL	0.9 mL	1:10

**Table 3:** Cytocompatibility profile of LNPs and LCNPs in HaCaT cells: cell viability percentage and half-maximal inhibitory concentrations (IC_50_, mg/mL). Cell viability and IC50 were measured by MTT assay. Cell viability values are reported as percentage viability of the control sample. Data are expressed as mean ± SD of three independent experiments. Statistical significance between the various experimental groups and the untreated control was determined using one-way analysis of variance (ANOVA), followed by Tukey post-hoc test (Table S1).

Nanoparticles Concentration/IC_50_, mg/mL	LNPs	LCNPs
**24 h**		
0.5	84.023 ± 1.182	85.352 ± 1.438
1	84.978 ± 0.983	87.608 ± 1.061
2	73.822 ± 0.531	81.767 ± 2.406
4	77.546 ± 1.225	82.141 ± 1.614
IC_50_	8.509 ± 0.221	12.260 ± 0.562
**48 h**		
0.5	81.528 ± 0.612	80.055 ± 3.874
1	81.191 ± 2.11	77.221 ± 3.354
2	73.185 ± 2.626	72.724 ± 1.514
4	66.035 ± 2.132	62.313 ± 1.745
IC_50_	5.880 ± 0.127	5.080 ± 0.094
**72 h**		
0.5	68.368 ± 0.991	78.119 ± 1.034
1	59.196 ± 1.111	63.369 ± 6.927
2	39.068 ± 0.541	59.988 ± 0.964
4	31.447 ± 1.092	56.631 ± 1.695
IC_50_	1.372 ± 0.021	3.054 ± 0.110

**Table 4:** Tukey’s post-hoc multiple comparison matrix following one-way ANOVA for lipid peroxidation, DPPH, and BMPO-based radical scavenging assays. Statistical significance was defined as *p* < 0.05 (values in bold).

**Lipid peroxidation, %**						
	5 min			*20 min*		
	PBN (control)	LNP	LCNP	PBN (control)	LNP	LCNPs
PBN (control)	1	<0.0001	<0.0001	1	<0.001	<0.001
LNP		1	0.0029		1	0.0061
LCNP			1			1
**DPPH scavenging activity, %**						
	5 min			*20 min*		
	DPPH (control)	LNPs	LCNPs	DPPH (control)	LNPs	LCNPs
DPPH (control)	1	<0.001	<0.001	1	<0.001	<0.001
LNPs		1	**<** **0.001**		1	0.073
LCNPs			1			1
**BMPO/•O_2_^−^ scavenging activity, %**						
	5 min			20 min		
	BMPO/•O_2_**^−^** (control)	LNPs	LCNPs	BMPO/•O_2_**^−^** (control)	LNPs	LCNPs
BMPO/•O_2_^−^ (control)	1	<0.001	<0.001	1	<0.001	<0.001
LNPs		1	0.0011		1	0.0001
LCNPs			1			1
**BMPO/CH_3_O^−^ scavenging activity, %**						
	5 min			20 min		
	BMPO/CH_3_O^−^ (control)	LNPs	LCNPs	BMPO/CH_3_O^−^ (control)	LNPs	LCNPs
BMPO/CH_3_O^−^ (control)	1	<0.001	<0.001	1	<0.001	<0.001
LNPs		1	0.0029		1	<0.001
LCNPs			1			1

**Table 5:** Minimum inhibitory concentrations (MIC) and minimal bactericidal concentrations (MBC) of lignin, lignin/chitosan solutions and LNPs and LCNPs nanosuspensions.

Minimum inhibitory concentration (MIC) of solutions, mg/L *		
	Lignin solution	Lignin + chitosan solution
*S. aureus ATCC 25923*	18,000.00	ND
*B. cereus*	4500.00	1125.00
*E. coli ATCC 25922*	18,000.00	ND
*Ps. aeruginosa ATCC 27853*	ND	ND
Minimum inhibitory concentration (MIC) of nanosuspensions, mg/L **		
	LNPs suspension	LCNPs suspension
*S. aureus ATCC 25923*	4550.00	1137.50
*B. cereus*	568.75	284.38
*E. coli ATCC 25922*	2275.00	1137.50
*Ps. aeruginosa ATCC 27853*	2275.00	1137.50
**Minimum bactericidal concentrations (MBC) of nanosuspensions, mg/L**		
	LNPs suspension	LCNPs suspension
*S. aureus ATCC 25923*	18,200.00	ND
*B. cereus*	2275.00	4550.00
*E. coli ATCC 25922*	4550.00	ND
*Ps. aeruginosa ATCC 27853*	18,200.00	ND

* Solutions concentration final range 18,000, 9000, 4500, 2250, 1125, 562.5 mg/L; ND = not determined at the highest tested concentration of 18,000 mg/L; Stock solution concentration—36,000 mg/L; Minimum bactericidal concentrations were not established within the tested range of 18,000–2250 mg/L. ** Stock suspension concentration: 36,400 mg/L, final concentrations—18,200; 9100; 4550; 2275; 1137.5; 568.75; 284.38; 142.19; 71.09; 35.55 mg/L. ND = not determined at the highest tested concentration of 18,200.00 mg/L.

## Data Availability

The original contributions presented in this study are included in the article/Supplementary Material. Further inquiries can be directed to the corresponding author.

## Supplementary Materials

The following supporting information can be downloaded at: https://www.mdpi.com/article/10.3390/pharmaceutics18030350/s1, Table S1: Tukey’s post-hoc multiple comparison analysis following one-way ANOVA for the biocompatibility profile of LNPs and LCNPs in HaCaT cells; Table S2: Tukey’s HSD post-hoc comparisons following one-way ANOVA for reducing power, DPPH, hydroxyl radical, ABTS, nitric oxide, and superoxide radical scavenging assays; Methods S3. Methods for synthesis if LNPs and LCNPs.
