# Supplementary material for: Comparative Biofunctionality Assessment of Lignin and Lignin/Chitosan Nanoparticles: Impact of Chitosan Co-Assembly on Cytotoxicity, Cytocompatibility, Radical-Scavenging Activity, and Antimicrobial Performance
Source: Pharmaceutics. 2026 Mar 11;18(3):350. doi: 10.3390/pharmaceutics18030350 (PMC13028907; doi:10.3390/pharmaceutics18030350)
Supplement: Supplementary file 1 [file pharmaceutics-18-00350-s001.zip › pharmaceutics-4184378-supplementary.pdf]

Table S1. Tukey's post-hoc multiple comparison analysis following one-way ANOVA for the biocompatibility profile of LNPs and LCNPs in HaCaT cells:. Statistical significance was defined as $p < 0.05$ (values in bold).

		**24 h**		
concentration	group	LNPs	LCNPs	control
0.5 mg/mL	LNPs	—	0.5165	**< 0.0001**
	LCNPs		—	**< 0.0001**
	control			—
1 mg/mL	LNPs	—	0.1626	**0.0001**
	LCNPs		—	**0.0003**
	control			—
2 mg/mL	LNPs	—	**0.0122**	**< 0.0001**
	LCNPs		—	**0.0002**
	control			—
4 mg/mL	LNPs	—	**0.0114**	0
	LCNPs		—	0
	control			—
		**48 h**		
0.5 mg/L	LNPs	—	0.9026	**0.0001**
	LCNPs		—	**0.0001**
	control			—
1 mg/L	LNPs	—	0.2137	**0.0005**
	LCNPs		—	**0.0001**
	Control			—
2 mg/L	LNPs	—	0.7567	**< 0.001**
	LCNPs		—	**< 0.001**
	control			—
4 mg/L	LNPs	—	0.156$	**< 0.001**
	LCNPs		—	**< 0.001**
	control			—
		**72 h**		
0.5 mg/L	LNPs	—	**0.0004**	**< 0.001**
	LCNPs		—	**< 0.001**
	control			—
1 mg/L	LNPs	—	0.3664	**< 0.001**
	LCNPs		—	**0.0001**
	control			—
2 mg/L	LNPs	—	**< 0.001**	**< 0.001**
	LCNPs		—	**< 0.001**
	control			—
4 mg/L	LNPs	—	**< 0.001**	**< 0.001**
	LCNPs		—	**< 0.001**
	control			—

Table S2. Tukey's HSD post-hoc comparisons following one-way ANOVA for reducing power, DPPH, hydroxyl radical, ABTS, nitric oxide, and superoxide radical scavenging assays. Statistical significance is indicated as follows: *p < 0.05; **p < 0.01; ***p < 0.001.

**Reducing power assay, %**			
	control	LNPs	LCNPs
control	1	< 0.001***	0.0001***
LNPs		1	0.0269*
LCNPs			1
**DPPH, %**			
control	1	< 0.001***	< 0.001***
LNPs		1	0.0629 (ns)
LCNPs			1
**Hydroxyl radical scavenging assay, %**			
control	1	< 0.001***	< 0.001***
LNPs		1	0.0648 (ns)
LCNPs			1
**ABTS radical scavenging activity, %**			
control	1	< 0.001***	< 0.001***
LNPs		1	0.0036**
LCNPs			1
**Nitric oxide scavenging assay, %**			
control	1	< 0.001***	< 0.001***
LNPs		1	< 0.001***
LCNPs			1
**Superoxide radical scavenging potential, %**			
control	1	< 0.001***	< 0.001***
LNPs		1	0.0387 *
LCNPs			1

S3 Methods for synthesis of LNPs and LCNPs

LNPs were synthesized using a solvent-shifting precipitation method [32]. Alkali lignin was dissolved in ultrapure water to obtain a 5 mg/mL solution, followed by the addition of 1 mL ethanol. The mixture was stirred at 500 rpm for 3 min to ensure complete dissolution. Nanoparticle formation was then induced by the dropwise addition of 7 mL of 1% (w/v) citric acid at an approximate rate of 4 mL/min under continuous stirring. The suspension was further stirred for 10 min to allow complete precipitation and stabilization of the particles. The resulting nanoparticle dispersion was centrifuged at 15,000 × g for 30 min at 10 °C. The collected particles were washed three times with ultrapure water to remove residual reagents. Subsequently, the suspension was ultrasonicated in an ice bath at 96% amplitude to improve dispersion and prevent aggregation. Finally, the nanoparticles were lyophilized at −64 °C.

LCNPs were prepared using a multistep synthesis process integrating solvent–antisolvent precipitation, self-assembly, ultrasonication, and freeze-drying [33]. Briefly, an aqueous alkaline lignin

solution containing 1 mL of 96% ethanol was used as the starting phase. Subsequently, 1 mL of 0.5% (w/v) chitosan solution prepared in 1% (v/v) lactic acid was added dropwise at a controlled flow rate under continuous stirring. The resulting suspension was then subjected to ultracentrifugation at 15,000 × g for 30 min at 10 °C using a Hermle Z 326 K ultracentrifuge (HERMLE Labortechnik GmbH, Wehingen, Germany). The collected nanoparticles were washed with ultrapure water (INTEGRITY+ ultrapure water system, Adrona, Latvia) to remove unbound components and residual solvents. In the next step, the nanoparticle suspension was ultrasonically homogenized using a Bandelin Sonopuls HD 2070 ultrasonic homogenizer (BANDELIN Electronic GmbH & Co. KG, Berlin, Germany) in an ice bath to prevent thermal degradation and ensure uniform particle dispersion. Finally, the homogenized nanoparticles were freeze-dried in a vacuum lyophilizer (Biobase Bioindustry Ltd., Jinan, China) at −64 °C to obtain dry blank LCNPs.

The obtained nanoparticles were stored in airtight containers protected from light and moisture at 4°C until further use.
